# Supplementary material for: E3 ligases RNF43 and ZNRF3 display differential specificity for endocytosis of Frizzled receptors
Source: Life Sci Alliance. 2024 Jul 8;7(9):e202402575. doi: 10.26508/lsa.202402575 (PMC11231576; doi:10.26508/lsa.202402575)
Supplement: Supplementary file 1 [file LSA-2024-02575_SdataF1.pdf]

Source file_Figure 1

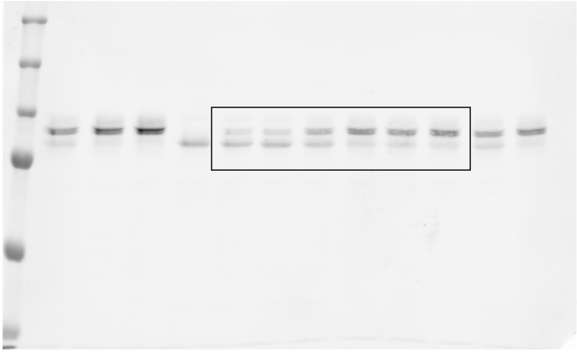

Figure 1B_rabbit anti-DVL2 (WT)

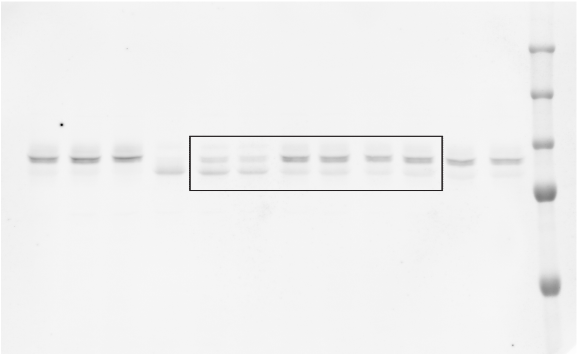

Figure 1B_rabbit anti-DVL2 (RZdKO)

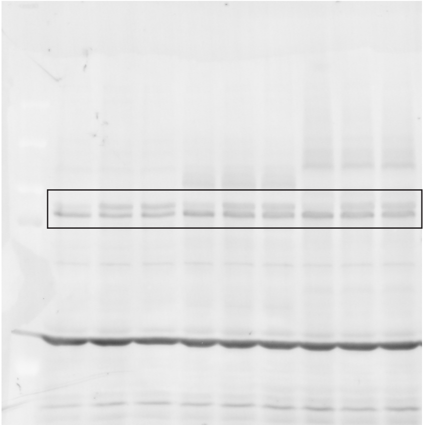

Figure 1C_rabbit anti-DVL2

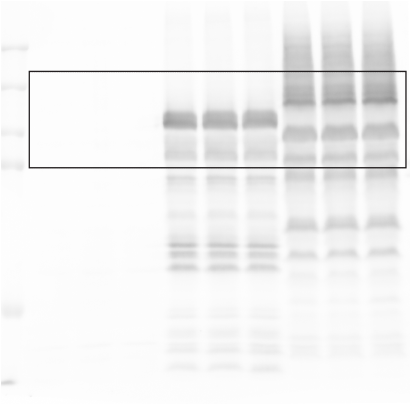

Figure 1C_rat anti-HA
