# Supplementary figures and images for: E3 ligases RNF43 and ZNRF3 display differential specificity for endocytosis of Frizzled receptors
Source: Life Sci Alliance. 2024 Jul 8;7(9):e202402575. doi: 10.26508/lsa.202402575 (PMC11231576; doi:10.26508/lsa.202402575)

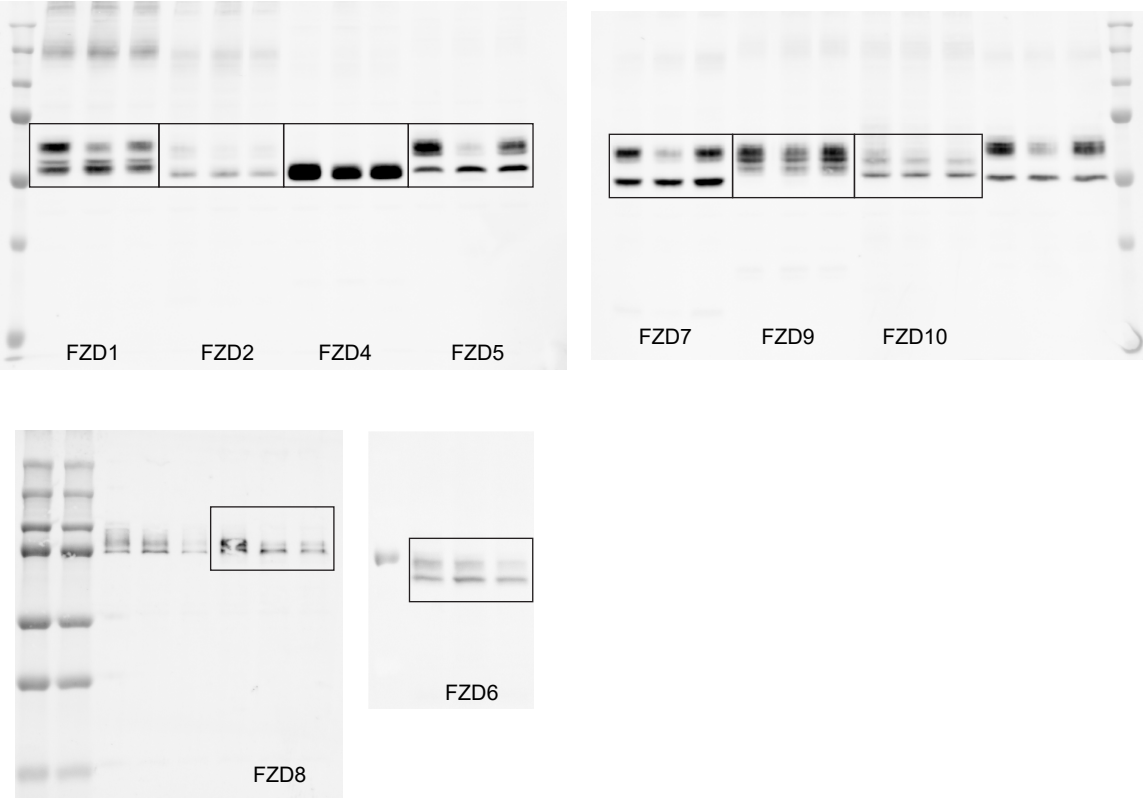

Figure 2A\_mouse anti-V5

Supplement: Supplementary file 2 [file LSA-2024-02575_SdataF2.pdf]
