# Supplementary material for: E3 ligases RNF43 and ZNRF3 display differential specificity for endocytosis of Frizzled receptors
Source: Life Sci Alliance. 2024 Jul 8;7(9):e202402575. doi: 10.26508/lsa.202402575 (PMC11231576; doi:10.26508/lsa.202402575)
Supplement: Supplementary file 3 [file LSA-2024-02575_SdataFS1_FS2.pdf]

Source file_Figure S1+2

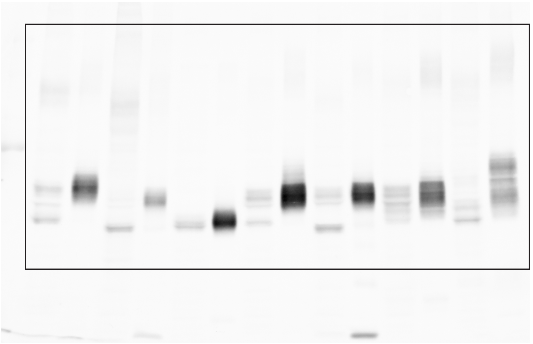

Figure S1A_mouse anti-V5

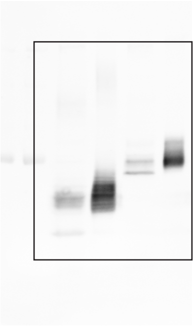

Figure S1A_mouse anti-V5

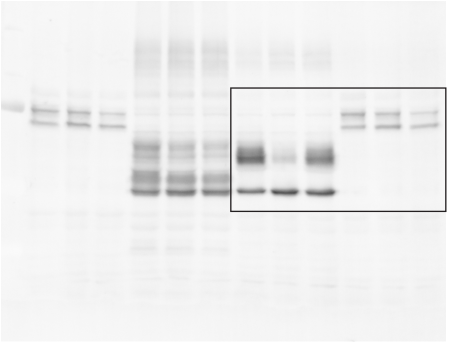

Figure S2D_mouse anti-V5

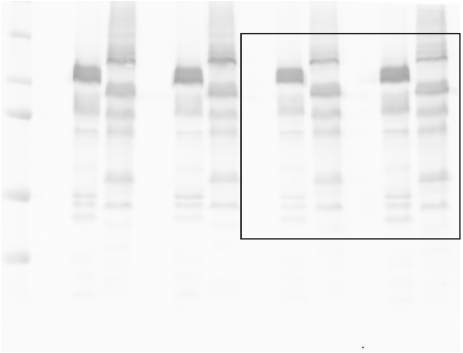

Figure S2D_rat anti-HA

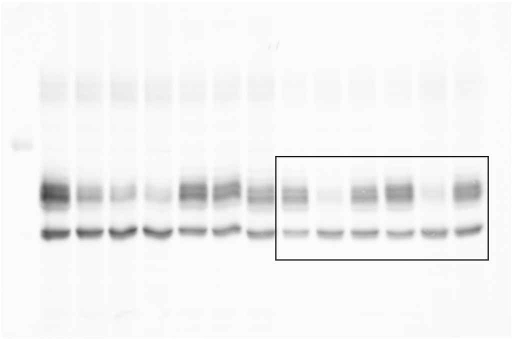

Figure S2E_mouse anti-V5

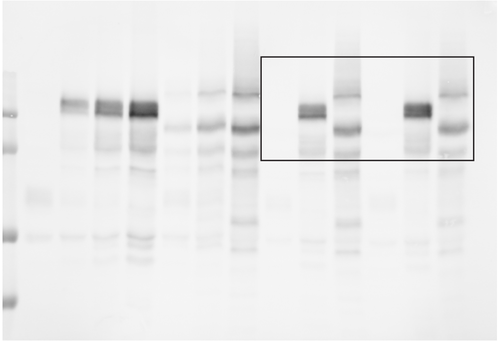

Figure S2E_rat anti-HA

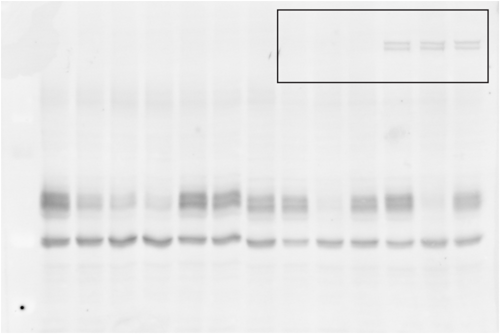

Figure S2E_rabbit anti-myc
