# Supplementary material for: E3 ligases RNF43 and ZNRF3 display differential specificity for endocytosis of Frizzled receptors
Source: Life Sci Alliance. 2024 Jul 8;7(9):e202402575. doi: 10.26508/lsa.202402575 (PMC11231576; doi:10.26508/lsa.202402575)
Supplement: Supplementary file 4 [file LSA-2024-02575_SdataF3.pdf]

Source file_Figure 3

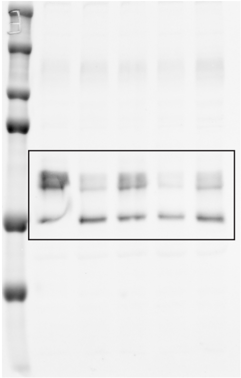

Figure 3A_mouse anti-V5

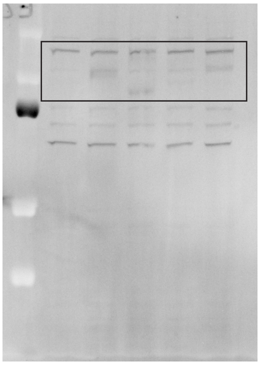

Figure 3A_rabbit anti-Flag

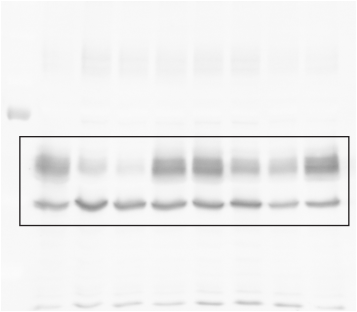

Figure 3D_mouse anti-V5

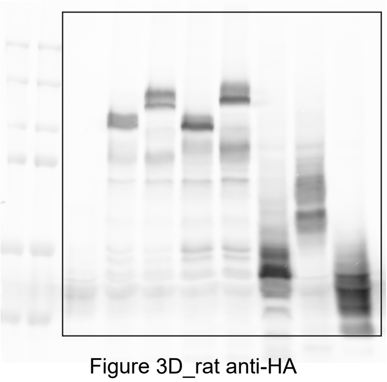

Figure 3D_rat anti-HA

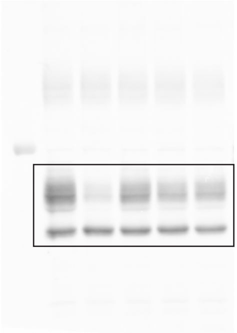

Figure 3E_mouse anti-V5

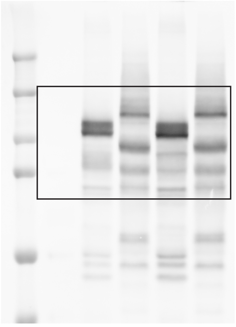

Figure 3E_rat anti-HA

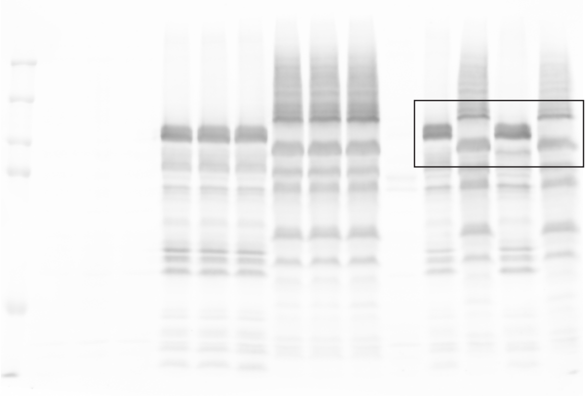

Figure 3F_mouse anti-V5

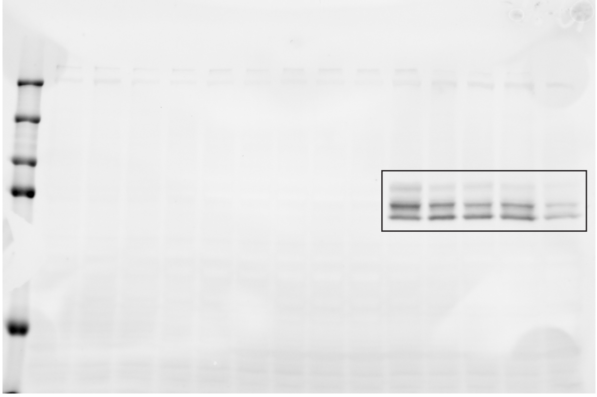

Figure 3F_rat anti-HA
